# A new approach to quantify visceral fat via bioelectrical impedance analysis and ultrasound compared to MRI

**DOI:** 10.1038/s41366-023-01400-7

**Published:** 2023-10-27

**Authors:** Jana Hoffmann, Jens Thiele, Stefan Kwast, Michael Andrew Borger, Thomas Schröter, Jochen Schmidt, Martin Busse

**Affiliations:** 1 Outpatient Clinic of Sports Medicine, University of Leipzig, Rosa-Luxemburg-Str. 20-30, 04103 Leipzig, Germany; 2 Department of Radiology, Helios Klinik, 04435 Schkeuditz, Germany; 3 Helios Health Institute, 13125 Berlin, Germany; 4 University Department of Cardiac Surgery, Heart Center, 04289 Leipzig, Germany; 5 Department of Neurophysics, Max Planck Institute for Human Cognitive and Brain Sciences, 04103 Leipzig, Germany

**Keywords:** Risk factors, Weight management, Endocrine system

## Abstract

**Background:**

Visceral adipose tissue (VAT) has been linked to systemic proinflammatory characteristics, and measuring it accurately usually requires sophisticated instruments. This study aimed to estimate VAT applying a simpler method that uses total subcutaneous fat and total body fat (BF) measurements.

**Method:**

As part of our experimental approach, the subcutaneous fat mass (SFT) was measured via US (SFT_total_), and VAT was quantified by assessing MRI data. Both parameters were added to obtain total body fat (BF_calc_). Those results were then compared to values obtained from a bioelectrical impedance analysis (BF_BIA_). Multiple regression analyses were employed to develop a simplified sex-specific equation for SFT, which was subsequently used in conjunction with BF_BIA_ to determine VAT (VAT_Eq_).

**Result:**

We observed excellent reliability between BF_BIA_ and BF_calc_, with no significant difference in body fat values (20.98 ± 8.36 kg vs. 21.08 ± 8.81 kg, *p* = 0.798, ICC 0.948). VAT_Eq_female/male_ revealed excellent reliability when compared to VAT_MRI,_ and no significant difference appeared (women: 0.03 ± 0.66 kg with a 95% CI ranging from −1.26 kg to 1.32 kg, *p* = 0.815, ICC: 0.955.; men: −0.01 ± 0.85 kg with a 95% CI ranging from −1.69 kg to 1.66 kg, *p* = 0.925, ICC: 0.952).

**Conclusion:**

Taking an experimental approach, VAT can be determined without MRI.

## Introduction

Overweight and obesity are associated with a range of chronic illnesses including cardiovascular disease, diabetes mellitus and cancer [1]; they are also strong predictors of increased mortality [2].

In recent decades, body fat distribution has become a major focus of research, as there is evidence that it is more important than total body fat mass (BF) in predicting obesity-related diseases [3]. The proportion of abdominal fat tissue comprising visceral (VAT) and subcutaneous adipose tissue (SAT) is a critical correlate for all health complications related to overweight and obesity [4, 5]. These two fat compartments are structurally and metabolically distinct [6, 7]. VAT is of particular interest due to its association with proinflammatory and angiogenic activation, including cytokine secretion such as IL-6 and other immune response regulators [7, 8]. It is also well-established that men tend to accumulate more VAT than SAT, while women tend to store more of the latter [9, 10]. Several studies have shown that weight loss through diet and exercise interventions triggers a significant reduction in VAT mass in obese individuals, partly due to catecholamine-induced lipolysis caused by greater β1- β3-adrenoreceptor density in visceral fat [7, 8, 11–15]. A reduction in VAT coincides with a drop in glucose, insulin, and leptin levels, contributing to a protective effect in relation to cardiovascular and metabolic conditions [8]. Given that VAT is known to contribute significantly to cluster of health conditions that augment the likelihood of developing heart disease, stroke, and diabetes, quantifying VAT precisely is extremely important. It is a parameter that could serve as a potential parameter for classifying an individual’s cardiovascular risk profile. Although reference methods for measuring VAT such as MRI, CT, and DXA exist, they are costly and time-consuming. Some studies have suggested applying single-slice MRI to determine VAT, but this method may not always accurately yield the total VAT mass [16–18]. Non-ionizing and cost-effective techniques such as abdominal bioelectrical impedance analysis (BIA) may be valuable for measuring total abdominal adipose tissue, but they are incapable of specifically quantifying visceral fat in comparison to MRI [19]. Moreover, while simpler methods like the waist-hip ratio (WHR) or waist circumference (WC) are frequently used, they do not specifically quantify VAT mass, as they provide information only on fat distribution [18, 20, 21]. The caliper is capable of assessing subcutaneous fat folds but is error-prone, particularly in the abdominal area [22]; it thus systematically underestimates the thickness of subcutaneous fat. Ultrasound (US) is a promising alternative thanks to its ability to quantify subcutaneous fat tissue and visualize visceral fat depth [23]. However, quantifying the total VAT mass is a complex task, and alternative practical and feasible methods suitable to a clinical setting have yet to be established.

In writing this paper we relied on the fundamental concept that the combined mass of subcutaneous fat (SFT) and VAT corresponds to total body fat when applying the “addition method”. By rearranging this formula, it becomes theoretically feasible to calculate VAT solely based on SFT and total BF measurements. BF is easily obtained through BIA in this context. Determining total SFT involves measuring the entirety of subcutaneous fat across the body via US and a previously implemented systematic mapping technique [22].

To enhance this procedure’s practicality, it would be additionally beneficial to utilize a simplified equation.

Three key points arise from this objective:

1. Will adding SFT and VAT align with to total BF determined by BIA?
2. Is it feasible to derive a simplified equation (SFT_Eq_) applying only three to four measuring points to accurately represent total SFT (SFT_total_)?
3. Can VAT be accurately determined by utilizing BF_BIA_ and SFT_Eq_ according to key point two?

## Subjects and methods

### Ethical aspects

The study was approved by the Ethics Committee of the Medical Faculty, University of Leipzig (097/17-ek, 089/18-ek) and followed the latest revision of the Declaration of Helsinki. All participants signed written informed consent forms and received an information letter.

### Participants

We enrolled 49 subjects aged a mean 49.98 ± 20.86 years in this study (Table 1). Based on BMI and BF, we included participants of normal weight and overweight to examine a wide range of body types. Study participants were excluded in case of pregnancy, any metal in the body, or any type of cardiac devices or leg edema (ie, due to heart failure).

**Table 1:** Descriptive data of sample.

	Total mean ± SD [*n* = 49]	Men mean ± SD [*n* = 24]	Women mean ± SD [*n* = 25]
Age [y]	49.98 ± 20.86	50.08 ± 22.18	49.88 ± 20.43
Height [cm]	170.86 ± 9.75	177.38 ± 7.77	164.60 ± 7.28
Weight [kg]	75.19 ± 11.53	80.82 ± 6.69	69.79 ± 10.91
BMI [kg/m^2^]	25.79 ± 3.69	25.75 ± 3.18	25.82 ± 4.26
BF [%]	27.57 ± 9.31	21.91 ± 6.67	33.01 ± 8.46
WHR	0.94 ± 0.09	0.97 ± 0.90	0.97 ± 0.09
WC [cm]	94.27 ± 12.67	95.88 ± 12.57	92.67 ± 13.09
HC [cm]	100.61 ± 8.66	98.71 ± 7.22	102.52 ± 9.85

*BF* body fat, *SD* standard deviation, *BMI* body mass index, *WHR* Waist-to-hip ratio, *WC* waist circumference, *HC* Hip circumference.

### Sample size

Our study is based on an experimental design. A total sample size of 41 was calculated using G-Power (Version 3.1.9.2) to achieve a power of 0.8 at a significance level of α < 0.05, with the ability to detect a difference of 10% (±22.5%) between measurements. To account for potential variations in body types, eight additional participants were included in the study taking an oversampling approach.

### Study design

Body fat measurements were taken within a single day. Prior to the examination, a medical history assessment was conducted and the participants’ weight, height and waist-to hip ratio (WHR) were assessed. SFT was then measured via US, total body fat was determined using bioelectrical impedance analysis (BIA), and we quantified visceral fat via magnetic resonance imaging (MRI). The measurements were taken in the morning, and participants were asked to fast overnight before measurements were taken. They were also instructed not to engage in any exercise or make any dietary changes the day before the measurements.

### Waist-to-hip ratio

As an additional anthropometric parameter, waist circumference (WC) was measured using a tape measure at the end of a normal expiration, specifically at the level of the lower floating rib. For hip circumference (HC), the tape was positioned around the hips at the level of the trochanter major and greatest gluteal protuberance. WHR was calculated by dividing WC by HC.

### Subcutaneous fat tissue measurement by ultrasound

To determine the SFT, the right side of the body was systematically divided into 56 rectangles, excluding head, hand, foot, and genital area. A previous study demonstrated the reliability of this mapping method [22]. The center of each rectangle was measured via US, and the length, width, depth, and fat density (0.949 g/cm^3^) [24] were multiplied to obtain the subcutaneous fat thickness. All width and length measurements were taken with an accuracy of 0.1 cm using a tape measure. The total subcutaneous fat mass on the right side of the body was obtained by adding together measurements from all 56 fields and doubling the measured SFT in the overall subcutaneous fat mass (SFT_total_). Detailed instructions were provided in a previous study [22].

After mapping, measurements were taken with the subject in supine position. US images were acquired using a B-Mode device (GE Healthcare GmbH, LOGIQ e, Vivid series). Depending on the approximate tissue depth, a 12 MHz linear transducer was used to measure SFT in longitudinal position. An optimum of brightness, gain and dynamic range was adjusted to improve tissue delineation. US gel was applied in the center of the field, with the probe placed longitudinally and perpendicularly to the tissue being examined, which revealed optimum echogenicity. When the boundaries were clearly visible, the US probe was lifted slowly until the ultrasound began to extinguish, applying the least amount of pressure possible. The area of interest was then frozen and measured from the beginning of the cutis to the muscle fascia. In the abdominal area, the image was taken when the subject stopped breathing at mid-tidal expiration. SFT was measured by an experienced scientific sonographer with five years of training.

### Bioelectrical Impedance analysis (BIA)

As human body tissues possess capacitive and resistive characteristics [25], this method relies on measuring resistance, which is then converted into total body water (TBW) predictions via an algorithm utilizing the relationship between volume, length, and resistivity. A resistivity value is assumed and included in the regression analysis to determine body composition [26]. Two skin electrodes are attached on each hand and foot for analysis, and the potential difference is measured. A constant electrical alternating current of 0.4 mA at 50 kHz is applied using a single frequency bioelectrical impedance device (AKERN BIA 101 Anniversary, AKERN-Srl, Florence, Italy). Free fat mass (FFM) and physiological fluids are good electrical conductors, while fat mass (FM) reacts with strong resistance. FM is obtained by subtracting FFM from weight. Although concerns have been raised about BIA’s accuracy, Ward et al. clarified that they are of comparable magnitude to the gold standard when performed under standardized conditions [26]. To ensure sufficient variability in VAT across the sample, a wide range of BMIs and body fat types was included in our study. The examination was conducted in the morning, and the bioelectrical impedance analysis (BIA) done immediately after the participants had spent two hours in recumbent position during the US measurement. We obtained BF parameters using BodyComposition V. 8.5 Professional Software (www.medi-cal.de). This software applies statistical analysis to determine the FFM hydration fraction instead of using the specified hydration level of 73.2% for a healthy population.

### MRI

A Philips Achieva 1.5 T MRI scanner was used in our study to acquire images of the abdomen. A whole-body coil was used to obtain sufficient imaging coverage in the peripheral regions of the homogeneous magnetic field. The system’s gradient strength was 33 mT/m with a maximum gradient slew rate of 122 T/m/s. Participants were examined in supine position and MRI imaging was done from the diaphragm to symphysis. Our MRI protocol is in Table 2.

**Table 2:** Abdominal MRI protocol.

Region	Sequence	Slice thickness (mm)	TR (ms)	TE (ms)	flip	time (min:sec)
Survey sagittal	GE	3	81	4.6	45	00:35
Survey transversal	GE	3	94	4.6	45	01:23
abdominal wall sagittal	SSh-TSE T2w	10	818	80	90	00:12
abdominal wall sagittal	TSE T1w	8	350	10	90	00:57
upper abdomen transversal	TSE T1w	8	350	10	90	00:15
upper abdomen transversal	SSh-TSE T2w	10	818	80	90	00:12
upper abdomen transversal	SSh-TSE T2w	8	818	80	90	0:12 (2x)
lower abdomen transversal	SSh-TSE T2w	8	818	80	90	0:12 (2x)

*T1w* T1-weighted, *T2w* T2-weighted, *GE* gradient echo-sequence, *Flip* Flipangle, *SSh-TSE* single shot-turbo spin echo-sequence, *Survey* planning sequence/fast gradient echo sequence, *BFFE* Fast field echo sequence, *TR* repetition time, *TE* echo time.

Imaging visceral adipose tissue (VAT) is challenging; motion artefacts caused by respiration, peristalsis and vascular pulsation, as well as ghost artefacts, must be minimized to ensure accurate delineation of adipose tissue from organs [27]. The abdomen and pelvis were measured during respiratory arrest. Compared to inspiration, the expiratory maneuver provides a well-defined respiratory position enhancing reproducibility [28]. Due to the longer echo time (TE), T2-weighted measurements produce lower flow artifacts (dark vessel lumen) [29]. This contributes to fewer ghost artifacts in phase direction (anterior-posterior). In addition, adipose tissue exhibits high signal intensity due to the long T1 and T2 values of fatty compounds, making fast T2-weighted sequences well-suited to define borders to the intestine and abdominal wall [29]. The short measurement time of under one second per slice reduces the impact of intestinal motility on image quality. Furthermore, motion artefacts are reduced on 2D slice acquisitions compared to a similar 3D sequence.

Hence, we measured abdomen and pelvis with a cross-sectional 2D slice selective single shot turbo spin-echo sequence (SSH-TSE). To ensure full coverage, acquisition was performed twice, for the lower and upper abdomen, respectively. Interleaved staircase acquisition was done within such a slab with 8 mm slices and 2 mm gaps. The reconstructed slices are taken correspondingly to cover 10 mm in slice direction in subsequent volume estimations. To cover the full slab, the acquisition was again subdivided into 2 runs. The interleaved staircase configuration ensured 10 mm slice and 10 mm gap alternations before using a 10 mm offset in the second run to yield the remaining gap. This setup minimizes cross-talk and magnetization transfer effects by prolonging the duration between the saturation of adjacent slices. The complete study resulted in 4 packages of 15 slices each, thus, 60 MRI slices were used to quantify VAT.

**Fig. 1:**
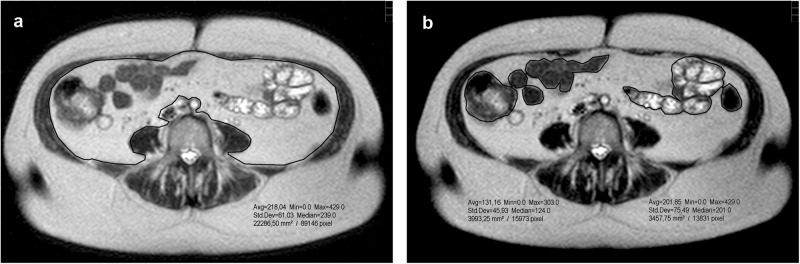
Polygonal traction measurement by PACS JiveX software. **a** Selection of the overall intraabdominal area, **b** Two-dimensional measurement of organs/tissues area.

We evaluated the images using the PACS JiveX software from Visus Health IT GmbH (www.visus.com). Each slice image was individually processed using the polygonal traction measurement software tool to determine total intra-abdominal area (Fig. 1a) and the boundaries between bowel structures (Fig. 1b) of SSH-TSE images. Both results were subtracted (cm^2^) and multiplied by 10 mm slice thickness and a density of 0.949 g/cm^3^ to obtain VAT mass. Our measurements showed excellent intra-rater (ICC 0.993, *p* = 0.778) and inter-rater (ICC 0.997, *p* = 0.263) reliability. T1 sequences were only used to help identify adipose tissue.

### Statistics

The initial stage of our data analysis involved comparing two techniques for measuring the overall amount of body fat: bioelectrical impedance analysis (BF_BIA_) and the “addition method” (BF_calc_). The addition method combines measurements of VAT acquired through MRI and the total subcutaneous fat measured using US (SFT_total_) across all fields (1–56).

In the next step, we simplified the procedure by developing a concise equation specifically for men and women (SFT_Eq_female/male_) to quantify total subcutaneous fat. This enabled us to identify representative locations for subcutaneous fat mass. This preliminary process laid the foundation for our subsequent quantification of VAT.

The third step comprised calculating VAT (VAT_Eq_female/male_) by employing the representative SFT locations obtained from SFT_Eq_female/male_ and the BF_BIA_ values in a multiple regression analysis. Finally, we compared VAT measured by MRI (VAT_MRI_) with VAT_Eq_female/male_ to assess the reliability and accuracy of these methods.

Statistical analysis was performed using GraphPad Prism 9.1.1 (GraphPad Software Inc., California, USA, www.graphpad.com) to calculate the mean, standard deviation (SD), Pearson’s correlation coefficient (r), and R_adj_ (adjusted R^2^). A two-sided paired t-test with a *p*-value (α < 0.05) and 95% confidence interval (CI), and a Bland-Altman plot [30] were used when differences between methods passed the normality test. Reliability was determined using the Intraclass coefficient (ICC) in SPSS 27 (SPSS Inc., Illinois, USA), with two-way mixed, single measures, absolute agreement. The ICC classifications were as follows: poor reliability (≤0.5), moderate reliability (>0.5–0.75), good reliability (>0.75–0.9), and excellent reliability (values greater than 0.90) [31].

To generate a simplified subcutaneous fat equation for men and women through regression analysis, the subcutaneous fat fields (1–56) were narrowed down using principal component analysis (PCA). Only 25 fields were integrated, as they revealed the highest intra- und interrater reliability [22] of each body part and were easily detectable via anatomical landmarks. Requirements for PCA use were tested in advance (e.g., normality, linearity, multicollinearity). This statistical analysis can only be used to reduce dimensions when variables correlate, therefore Bartlett’s sphericity test gives an indication of the correlation’s strength with significant differences (*p* < 0.05) indicating that PCA can be used for the data [32, 33]. The F-test in PCA compares the variance between components to the variance within components [34]. A statistically significant F-value (i.e., a high value) indicates that at least one principal component differs significantly from the others, and can explain a significant proportion of the variance in the data set [34]. We then ran the Kayser-Meyer-Olkin test (KMO) to test our sample’s adequacy, measuring the proportion of variance between variables attributable to common variance [35]. A KMO value of at least 0.6 is required to ensure an adequate sample [33]. To generate the number of factors/components (i.e. arm, leg, abdomen), we relied on the number above the eigenvalue of one [36]. The eigenvalue describes how much variance can be explained by the factor [37]. The resulting component matrix illustrates the factors’ loadings, which should be at least >0.5, indicating to degree to which a given factor contributes to explaining the variable across all individuals [38]. A rotation matrix is then generated via VariMax Rotation to analytically ensure that certain variables per factor load high and others load low [39]. Finally, two values exhibiting high loadings for each component were selected for multiple regression analysis. We took a stepwise approach using the SFT_Eq_ to identify suitable measurement locations. The inclusion variant was employed in the VAT_Eq_ to integrate fixed parameters, such as SFT and BF_BIA_ parameters. In the case of males, waist circumference (WC) was further incorporated into the equation, leading to enhanced outcomes. Conversely, for women, hip circumference (HC) was included in the VAT equation to offer a more comprehensive explanation of the proportionate variance and a lower 95% CI.

## Results

Our comparison of total body fat methods and VAT results are shown in Table 3 with their corresponding ICC and *p*-value. Sex-specific data are also integrated.

**Table 3:** SFT and VAT values determined via BIA, US, MRI and regression analysis.

Variable [kg]	Mean ± SD	*p*-value	*r*	R^2^ (R_adj_^2^)	ICC
Total sample [*n* = 49]					
BF_BIA_	20.98 ± 8.36	0.798	0.948	0.899	0.948
BF_calc_ (SFT_total_ +VAT_MRI_)	21.08 ± 8.81				
SFT_total_	17.67 ± 7.50				
VAT_MRI_	3.41 ± 2.50				
Male [*n* = 24]					
BF_BIA_	18.09 ± 7.02	0.257	0.900	0.812	0.899
BF_calc_ (SFT_total_ +VAT_MRI_)	17.35 ± 6.93				
SFT_total_	13.50 ± 4.62	0.995	0.977	0.955 (0.945)	0.978
SFT_Eq_male_	13.50 ± 4.51				
VAT_Eq_male_	3.87 ± 2.72	0.925	0.954	0.899 (0.871)	0.955
VAT_MRI_	3.85 ± 2.85				
Female [*n* = 25]					
BF_BIA_	23.75 ± 8.90	0.056	0.939	0.881	0.965
BF_calc_ (SFT_total_ +VAT_MRI_)	24.66 ± 9.22				
SFT_total_	21.68 ± 7.75	>0.999	0.975	0.951 (0.944)	0.976
SFT_Eq_female_	21.68 ± 7.56				
VAT_Eq_female_	3.06 ± 2.03	0.815	0.951	0.904 (0.878)	0.952
VAT_MRI_	2.98 ± 2.15				

*ICC* Intraclass coefficient, *SD* standard deviation, *BF*_*calc*_ the sum of total subcutaneous fat tissue plus visceral adipose tissue obtained via MRI, *VAT*_*MRI*_ visceral adipose tissue measured by MRI, *BF*_*BIA*_ total body fat determined via bioelectrical impedance analysis, *VAT* visceral adipose tissue, *MRI* magnetic resonance imaging, *SD* standard deviation, *VAT*_*Eq_female/male*_ visceral adipose tissue calculated via visceral adipose tissue Eqs. (3) and (4) for men or women, *SFT*_*total*_ total subcutaneous fat measured by ultrasound, *SFT*_*Eq_female/male*_ subcutaneous fat determined via Eqs. (1) and (2) for men or women, *R*_*adj*_^*2*^ adjusted R-squared of linear model, *R*^*2*^ R-squared.

### Total body fat: BIA vs. Addition method (BF_calc_ = SFT_total_ + VAT_MRI_)

**Fig. 2:**
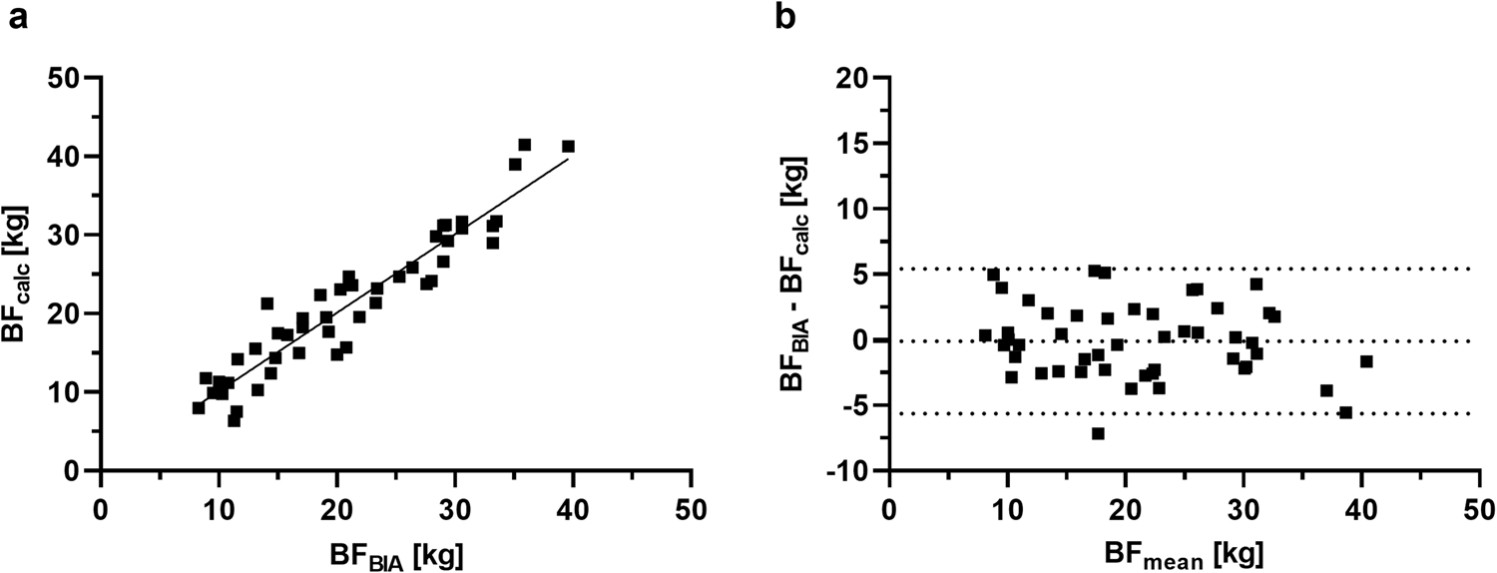
Correlation and Bland-Altman plot of BF_calc_ and BF_BIA_. **a** Correlation of total body fat measured by BIA (BF_BIA_) and calculated via addition of SFT_total_ and VAT_MRI_ (=BF_calc_), Y = 0.998*X + 0.127. **b** Bland-Altman plot of BF_BIA_ and BF_calc_ showing a mean difference of −0.10 ± 2.82 kg with lower and upper 95% CI −5.63 to 5.42 kg of the total sample.

A strong correlation is evident between BF_BIA_ and BF_calc_ (*r* = 0.948) (Fig. 2a) for our total sample. Furthermore, a paired *t*-test (α < 0.05) reveals no significant difference between instruments, and excellent reliability is observed for BF_calc_ compared to BF_BIA_ (*p* = 0.798, ICC = 0.948). Figure 2b visualizes the associated Bland-Altman plot of BF_BIA_ and BF_calc_ displaying a mean of difference of −0.10 ± 2.82 kg with the 95% confidence interval (CI) values of −5.63 to 5.42 kg.

### Principal component analysis (PCA)

To select the most accurate fields for an SFT equation, PCA was performed as described. Our female sample’s PCA showed significant and sufficient sphericity according to Bartlett’s test (χ^2^ = 452.70) and a KMO measure of sample adequacy (0.708). Three components were identified which explained 78.73% of the total variance of the variables. Two variables with the highest loading of each component were used for stepwise multiple linear regression analysis, after confirming the requirements for this analysis (e.g., normality, linearity etc.). Three fields of the female sample exhibited significant influence on the subcutaneous fat mass, *F*(3,21) = 137.11, *p* < 0.001, *n* = 25. This is statistically significant, and suggests that the three principal components can explain a part of variance in the data set. Similarly, our male sample’s PCA revealed significant and sufficient sphericity (Bartlett’s test: χ^2^ = 333.05) and a KMO measure of sample adequacy (0.71). We identified four components that explained 83.86% of the total variance of all variables. We demonstrate that four fields of the male sample revealed significant influence on the subcutaneous fat mass, *F*(3,20) = 59.54, *p* < 0.001, *n* = 24.

### Subcutanous fat: SFT_total_ vs. SFT_Eq_

**Fig. 3:**
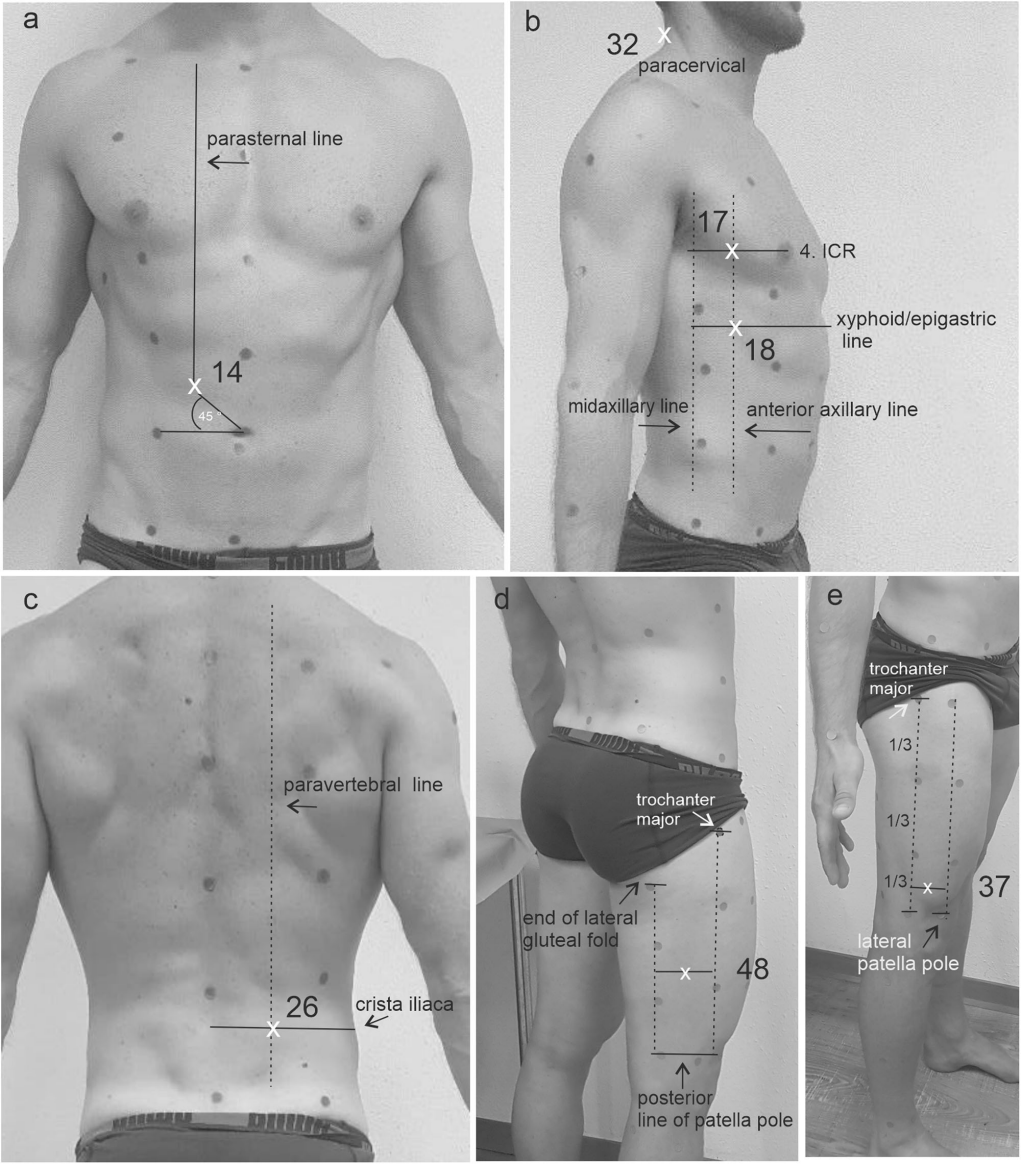
VAT_Eq_ landmarks. **a** Intersection of parasternal line and a 45°line at umbilicus creates F14. **b** Paracervical landmark at midcervical spinal (F32); Intersection of anterior axillar line with horizontal 4. ICR line (F17) and xyphoid/epigastric line (F18). **c** Intersection between paravertebral line and posterior, horizontal line of cristia iliaca. **d** One orientation point is identified at half distance of the vertical line from end of lateral gluteal fold to the horizontal posterior upper patella pole line. This orientation point creates a horizontal line ending at the vertical trochanter major line, where F48 is identified at the center. **e** The distance of vertical trochanter major line ending at the posterior patella pole line forms three equal sizes or lengths. The last third constitutes a point in its center, which is horizontally extended to the vertical patella pole line. The center of that line forms the measurement point (F37). All landmarks are designed for the body’s right side.

In our male participants, we found that three independent variables (fields 14, 18, and 48 in cm) included in the SFT Eq. (1) accounted for 94.4% of the total variance, demonstrating a strong effect size (Cohen’s f^2^ = 15.67). Similarly, four independent variables (fields 17, 37, 32, and 26 in cm) presented in the SFT Eq. (2) in women explained 94.5% of the total variance, exhibiting a strong effect size (Cohen’s f^2^ = 17.18) compared to SFT_total_. We detected no significant difference between SFT_Eq___female/male_ and SFT_total_, indicating excellent correlation and reliability (refer to Table 3). Figure 3 illustrates all landmarks visually for SFT_Eq_.

1 $$\begin{array}{ll}{SF}{T}_{{Eq}{{\_}}{female}}\left({kg}\right)=0.561+F18\,\left({lateral}\,{abdomen}\right)* 4.819\\\qquad\qquad\qquad\qquad\quad+\,F48\left({mid}\,{lateral}\,{posterior}\,{thigh}\right)* 4.462\\\qquad\qquad\qquad\qquad\quad+\,F14\left({umbilical}\right)* 1.589\end{array}$$

2 $$\begin{array}{ll}{SF}{T}_{{Eq}{{\_}}{male}}\left({kg}\right)=2.638+2.153* F17\left({lateral}\,{breast}\right)\\\qquad\qquad\qquad\qquad+\,6.132* F37\,\left({lower}\,{lateral}\,{thigh}\right)\\\qquad\qquad\qquad\qquad+\,1.311* F32\,\left({posterior}\,{neck}\right)\\\qquad\qquad\qquad\qquad+\,2.040* F26\,({lower}\,{back})\end{array}$$

### Visceral fat: VAT_MRI_ vs. VAT_Eq_

We included variables from BIA, the SFT_Eq_ fields, and WC and HC in a linear multiple regression model to arrive at an equation to estimate VAT in both men and women (refer to Eqs. (3) and (4)). Statistical analysis revealed that WC demonstrated significant benefits only for men, and HC for women. The inclusion variant multiple regression analysis revealed a VAT_Eq_female_ (3) explaining 87.1% of variance for men (Cohen’s f^2^ = 7.20) and 87.8% (4) for women (Cohen’s f^2^ = 7.20).

**Fig. 4:**
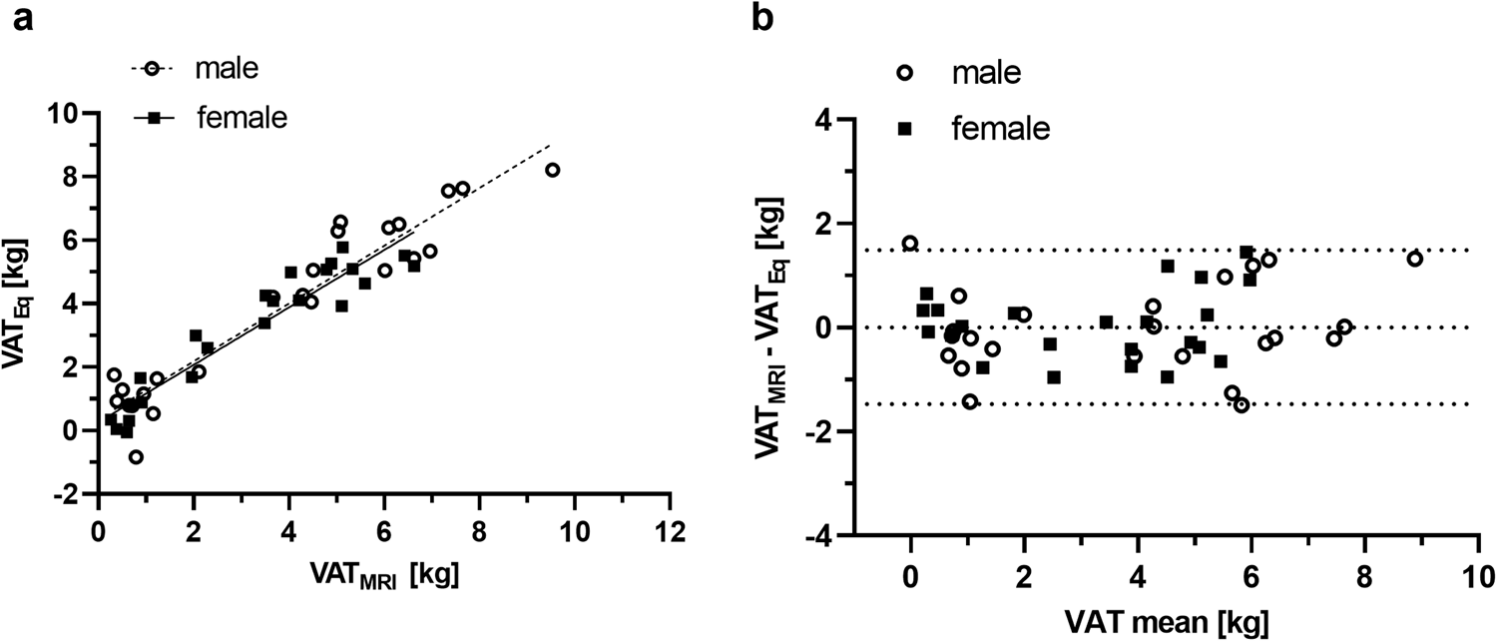
Sex-specific correlation and Bland-Altman plot of VAT_MRI_ and VAT_Eq_. (**a**) Correlation of VAT_Eq_male/female_ compared to VAT_MRI_: Y_male_ = 0.913*X + 0.652; Y_female_ = 0.849*X + 0.458 (**b**) Bland-Altman plot of VAT_MRI_ and VAT _Eq_male/female_ show a mean of difference of 0.03 ± 0.66 kg for women and −0.01 ± 0.85 kg for men. This graph shows upper and lower 95% CI of −1.47 kg up to 1.48 kg of total sample.

Comparing VAT_Eq_ values with MRI showed a strong positive correlation, as depicted in Fig. 4a and Table 3 for both men and women. Our findings also demonstrate excellent reliability. The average difference was 0.03 ± 0.66 kg with a 95% confidence interval (CI) ranging from −1.26 kg to 1.32 kg for women, and −0.01 ± 0.85 kg with a 95% CI ranging from −1.69 kg to 1.66 kg for men, as illustrated in Fig. 4b.

3 $$\begin{array}{l}{{\boldsymbol{VAT}}}_{{\boldsymbol{Eq}}{{\_}}_{{\boldsymbol{female}}}}({kg})={{\rm{BF}}}_{{\rm{BIA}}}({kg})* 0.139\\\qquad\qquad\qquad\qquad\quad+\,F14\,\left({cm}\right)* 0.587-F18\left({cm}\right)* 0.983\\\qquad\qquad\qquad\qquad\quad-\,F48\left({cm}\right)* 1.802+{HC}({cm})* 0.109-7.758\end{array}$$

4 $$\begin{array}{ll}{{\boldsymbol{VAT}}}_{{\boldsymbol{Eq}}{{\_}}{\boldsymbol{male}}}\left({kg}\right)=B{F}_{{BIA}}\left({kg}\right)* 0.144\\\qquad\qquad\qquad\qquad\quad+\,F17\left({cm}\right)* 0.302+F26\left({cm}\right)* 0.073\\\qquad\qquad\qquad\qquad\quad-\,F32\left({cm}\right)* 0.730-F37({cm})* 1.763\\\qquad\qquad\qquad\qquad\quad+\,{WC}({cm})* 0.151-11.502\end{array}$$

## Discussion

Our study demonstrates that total BF measurements obtained through BIA and the addition method delivered highly reliable results between the two methods. We also succeeded in developing simplified sex-specific equations which accurately estimated the total SFT yielded by the mapping method (SFT_total_). These excellent reliability results indicate that VAT can be determined without requiring MRI.

### Total Body fat: BF_BIA_ vs. Addition method (BF_calc_ = SFT_total_ + VAT_MRI_)

As we describe the addition procedure here for the first time, there is no other (published) data for comparison. Considering the Bland-Altman plot of total body fat measurement methods, both BIA and the addition method revealed a favorable mean difference. The lower and upper 95% confidence interval of −5.63 to 5.42 kg is a relatively large range. However, the absolute deviation of total body fat between methods remains consistent across our sample, resulting in a decrease in relative error with increasing total body fat. Bland-Altman plots help visualize differences, but they do not show whether the observed limits are clinically acceptable, as they depend on clinical need [32]. Johnsen et al. demonstrated that BIA and US correlate equivalently with total BF, suggesting that a combination of methods is generally feasible thanks to their equal sensitivity [40]. Our study confirms excellent reliability and correlation between BF_BIA_ and BF_calc_. Froelich et al. reported similar fat-mass values when determined by BIA and MRI (23.3 ± 10.9 kg and 22.7 ± 9.9 kg), but did not test for absolute agreement [18]. Our results acquired through BIA, US, and MRI measurements confirm their findings.

Regarding our sex-biased results, the relative mean of the difference between male BF_BIA_ and BF_calc_ was 8.13% (±23.79%) and female −3.58% (±9.85%). Although BIA’s fat mass was slightly higher in men and lower in women compared to BF_calc_, that difference was not significant, and the two methods demonstrated excellent reliability. This is evidence that sex is no limitation when applying these methods. Alicandro et al. reported excellent reliability in FFM for men and women when comparing BIA to DXA (ICC: male 0.95; female 0.89) [41], since DXA is considered the gold standard for assessing total fat mass.

In conclusion: when compared to BIA, the addition method for assessing total body fat utilizing SFT_total_ and VAT_MR_ fulfills the prerequisite for simplifying the SFT measuring process.

### Subcutaneous fat: SFT_total_ vs. SFT_Eq_

Two distinct equations for estimating SFT have been derived using PCA and multiple regression analysis. The reliability of these equations is remarkably high, and there are no noteworthy inconsistencies between SFT_total_ and the SFT estimates for female and male individuals (SFT_Eq_female_/_male_). It is therefore justifiable to utilize these equations to determine subcutaneous fat, and the precise locations the formula incorporates can be employed to calculate VAT. There is no alternative study of which we are aware that assesses SFT applying the same methodology.

The equations we describe account for different landmarks based on sex, with the lateral abdomen, mid lateral posterior thigh and umbilical area included for women and lateral breast, lower lateral thigh, lower back and posterior neck for men. Agrawal et al. confirmed significant sex-dimorphism in body fat distribution [42], highlighting the need for sex-specific equations when assessing SFT.

Compared to other body fat equations, our calculations differ in the output variable. While most calculate body density (BD) over the subcutaneous skinfold thickness, our formula specifically calculates the SFT mass. However, there is some similarity with the 3-factor formula of Jackson & Pollock in the location of such measurements [43, 44]. The known landmarks for Jackson & Pollock’s formula include the abdomen (near the navel), mid-anterior thigh, and lateral chest. While our locations may differ slightly, they cover a similar area. Nevill et al. [45] adapted Jackson & Pollock’s formula again, but their positions remained the same. In contrast, Durning and Womersely [46] included biceps, triceps, scapula and iliac crest in their body fat equation, with only the iliac crest bearing a slight resemblance to our landmark positions. Goran et al. [47] and Leahy et al. [48] added the calf to their formula. The development of these formulas and their correlation with total fat highlight the sensitivity of the sites. However, note that they employ a double-indirect approach: Although those particular sites presumably represent total SFT, which in turn correlates with total fat, they have not been specifically validated for total subcutaneous fat as an initial step. To reduce this gap, we take into account the measurement of total SFT. Most equations rely on caliper measurements which require caution as the caliper can vary from the reference depending on the body fat mass. Our equation is only applicable to our population and cannot yet be generalized without further verification studies.

### Visceral fat: VAT_MRI_ vs. VAT_Eq_

Regarding VAT_Eq_ for men and women, the 95% confidence intervals (CIs) are significantly lower than those of the addition method, indicating their applicability within an acceptable mean range of ±1.4 kg. Furthermore, the excellent reliability supports this conclusion. In addition to SFT_total_ and BIA parameters, anthropometric parameters (WC and HC) were included in the respective VAT_Eq_ to improve its validity. Given that VAT correlates positively with a higher WHR, it is unsurprising that our VAT_Eq_male_ model incorporates WC. Our investigation has also provided evidence supporting the notion that men exhibit higher VAT levels than women (3.85 kg vs. 2.96 kg, *p* < 0.016). Onat et al. revealed higher VAT in men as well [49]. Rantalainen et al. suggested that VAT may be influenced by sex-specific mRNA and miRNA expression in abdominal and gluteal adipose tissues [50]. Including either WHR or WC for men in the VAT equation contributed similarly to the proportion of the variance. Including these parameters resulted in an approximate 5% increase in adjusted R^2^ compared to the equation without WC or WHR and a lower 95% CI. Considering that WHR incorporates multiple measurements, we opted for WC as an additional parameter in our formula. Some studies have also indicated that WC is a better VAT indicator than WHR [49, 51–53]. We were able to confirm those findings while also observing that HC and VAT exhibited the weakest correlation in men (*r* = 0.689). Conversely, women showed a 6% higher adjusted R^2^ and a lower 95% CI between methods when HC was included, compared to WHR or WC alone, or without additional parameters. HC exhibited a highly positive correlation with SFT (*r* = 0.801) and VAT (*r* = 0.887) in women.

When considering VAT_Eq_ parameters, HC and WC are more heavily weighted within the formula than are subcutaneous fat depths and BIA because of their high values (e.g., 100 cm). This means that more technically demanding methods such as BIA and US do not contribute as strongly to the formula as do simple circumference measurement.

The Bland-Altman plot, similar to the comparison with total body fat methods, demonstrates consistent absolute deviation between methods across our total sample. This leads to a reduction in relative error as VAT increases. These findings suggest that the VAT formula is indeed applicable for overweight male and female adults.

### Conclusion

Our approach employing BIA and US may function as an initial step towards determining total VAT without MRI, DXA or CT. The following steps describe its application for practical use:

1. Assessing WC in males and HC in females.
2. Measuring subcutaneous fat depth at sex-specific landmarks via ultrasound.
3. Applying single-frequency BIA with two electrodes on each hand and foot.
4. Insert the obtained results into the sex-specific VAT formula.

### Outlook

With the rising global prevalence of obesity and related health conditions, there is growing awareness of the detrimental effects of excess visceral fat. Numerous innovative devices have been implemented to precisely measure visceral fat; but they are often time-consuming and expensive. As an experimental approach, our method can be employed to simplify the quantification of VAT and incorporate it as an additional parameter when assessing cardiovascular risk profiles. Further investigations, including the implementation of multifrequency BIA and its practical use in clinical setting, are necessary to refine this methodology.

### Limitation

Certain areas, such as the hands, feet, head, and genitals, were neglected, even though they may contain a small amount of subcutaneous fat. Our study cohort consisted only of young, normal-weight and older, overweight individuals. As young, normal-weight individuals have minimal visceral adipose tissue, the VAT_Eq_ primarily applies to overweight individuals. Further studies with larger samples and more diverse body types are necessary to validate these results. Our findings only apply to this study population and rely on fasting adults with diverse body types measured by a single frequency bioelectrical impedance analysis, thus they cannot be extrapolated to multifrequency BIA.

## Data Availability

The authors confirm that the data supporting the findings of this study are available within the article; further inquiries can be directed to the corresponding authors.
